# Fiber Optic Train Monitoring with Distributed Acoustic Sensing: Conventional and Neural Network Data Analysis

**DOI:** 10.3390/s20020450

**Published:** 2020-01-13

**Authors:** Stefan Kowarik, Maria-Teresa Hussels, Sebastian Chruscicki, Sven Münzenberger, Andy Lämmerhirt, Patrick Pohl, Max Schubert

**Affiliations:** 1 Bundesanstalt für Materialforschung und -prüfung (BAM), Unter den Eichen 87, 12205 Berlin, Germanymaria.hussels@bam.de (M.-T.H.); Sven.Muenzenberger@bam.de (S.M.); 2 DB Netz AG, Mainzer Landstr. 199, 60326 Frankfurt, Germany; Andy.Laemmerhirt@deutschebahn.com (A.L.); Patrick.Pa.Pohl@deutschebahn.com (P.P.); max.schubert@deutschebahn.com (M.S.)

**Keywords:** distributed fiber optic sensing, distributed acoustic sensing, train tracking, artificial neural networks

## Abstract

Distributed acoustic sensing (DAS) over tens of kilometers of fiber optic cables is well-suited for monitoring extended railway infrastructures. As DAS produces large, noisy datasets, it is important to optimize algorithms for precise tracking of train position, speed, and the number of train cars. The purpose of this study is to compare different data analysis strategies and the resulting parameter uncertainties. We present data of an ICE 4 train of the Deutsche Bahn AG, which was recorded with a commercial DAS system. We localize the train signal in the data either along the temporal or spatial direction, and a similar velocity standard deviation of less than 5 km/h for a train moving at 160 km/h is found for both analysis methods. The data can be further enhanced by peak finding as well as faster and more flexible neural network algorithms. Then, individual noise peaks due to bogie clusters become visible and individual train cars can be counted. From the time between bogie signals, the velocity can also be determined with a lower standard deviation of 0.8 km/h. The analysis methods presented here will help to establish routines for near real-time train tracking and train integrity analysis.

## 1. Introduction

Distributed acoustic sensing (DAS) is a powerful fiber optic technique that can detect vibrations with a resolution of a few meters along a standard telecom glass fiber many tens of kilometers long. With these unique and still improving capabilities, DAS is increasingly used in applications such as intrusion detection along a perimeter, leak monitoring along pipelines, monitoring of sub-sea cables, or seismic monitoring [1,2,3]. In these examples, the long-range sensing capabilities with a single fiber are used. Similarly, extended railway infrastructure is well-suited for DAS monitoring. By detecting the noise and vibrations caused by the train, it is possible to locate the train position, its velocity [4,5], and also its length, which may be important for confirming that no train cars have been decoupled. Thereby DAS may help to increase railway capacity by enabling efficient train driving and disposition, but more importantly by enabling moving block operation, if the safety-critical performance of DAS can be established. Further, not only can the train position and velocity be monitored, but, in principle, also faults of the train such as flat wheels, and wear in the train track can be detected due to their characteristic acoustic signatures [6].

While DAS is not yet routinely used in train monitoring, it offers several advantages over established techniques such as a track circuit or wheel counters. In contrast to wheel counters at a limited number of positions, fiber optic sensing is a truly distributed measurement and monitors the position of the train at all times. With its sensing range of tens or even up to 100 km [6] it eliminates the necessity of power and data cables because a single telecom fiber that often is already installed next to tracks can serve both, as sensor and data line. The DAS fiber is also immune to electromagnetic interference or lightning strike. In comparison to global navigation satellite systems, fiber sensing has the advantage of providing signals in tunnels and it does not need wireless communication between trains and base stations. Beyond train monitoring, events such as trespassing or cable theft can be detected with DAS. However, significant challenges remain before DAS can be implemented in routine railway operations.

A range of fiber optic DAS systems have been described in recent literature and significant progress has been made with respect to signal quality and sensing range. Fiber optic sensing techniques such strain sensors based on Brillouin scattering [7,8], fiber Bragg gratings and fiber interferometers [9,10] have been demonstrated in railway applications. However, most activities in the field of DAS have centered on Raleigh backscatter based (C-OTDR) systems [6,7,11,12]. Two main DAS techniques can be distinguished. On the one hand, there are simpler systems which only detect the vibration frequency but not the true signal amplitude or phase of the acoustic signal. On the other hand, there exist ‘true-phase’ DAS systems that enable quantitative measurement of the vibration and strain amplitude of the sensor fiber. Simpler DAS systems have been successfully used in a range of publications [4,5,7] and are also used in this work. Recent true-phase systems have shown significantly better signal-to-noise ratios as well as a long sensing range beyond 80 km [6]. A common problem of both DAS and true-phase DAS systems is the large amount of raw data that is acquired and must be processed precisely and quickly enough to extract the features of interest from the noisy data. Due to the random arrangement of Rayleigh scattering centers in the fiber, there is significant variability in signal strength and even partial fading of the signal for certain ranges. There is also significant temporal drift in the DAS data even though novel setups can produce stable signals at the cost of sampling frequency and range [13].

Significant filtering and data processing must be performed to extract the train position, velocity, and axle or bogie count from DAS data. A range of algorithms has been used in literature, such as high pass filtering, to remove slower signal drift; wavelet transforms to get cleaner train signals; or Canny and sliding variance edge detection to determine the leading and trailing edge of the train [14,15]. To successfully operate real-time monitoring systems in the future, the processing must be fast enough and capable of handling variable conditions of the signal, e.g., due to temperature fluctuations, changing permanent strain on the fiber, or interfering background traffic noise. Apart from conventional deterministic algorithms, a promising route to enable fast analysis of DAS signals are artificial neural networks (ANN). ANN have been applied for pattern recognition and classification of events such as pedestrians or construction work next to tracks [16,17] but can also speed up the processing of DAS raw data treatment [18]. 

In this work, we present optimized conventional and artificial neural network algorithms and quantify the precision that can be achieved in train monitoring. 

## 2. Methods

**Figure 1:**
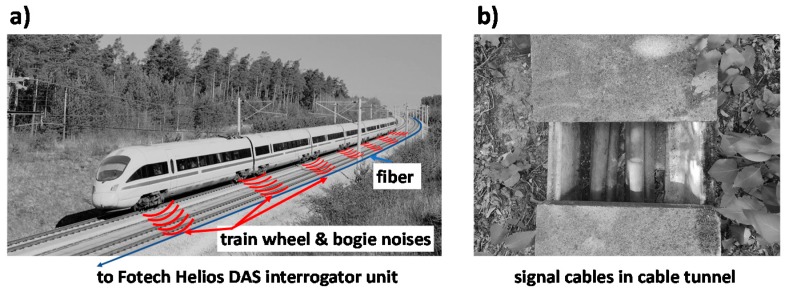
(**a**) Schematic of ICE train on ballastless track emitting noises that are picked up by a fiber optic distributed acoustic sensing (DAS) interrogator. (**b**) Standard telecom signal cables lying within cable tunnels are used for sensing.

The measurements were performed on a 35 km stretch of the ICE fast train track (see Figure 1a) between Erfurt and Halle (Verkehrsprojekt Deutsche Einheit Nr. 8.2 (VDE 8.2)). This railway line is newly built and therefore sensing conditions are homogeneous along this track. A standard telecommunication single-mode fiber lying in a trough next to the rail tracks (see Figure 1b) was used as the sensor element. The DAS signals of such a fiber are not as strong as for fibers affixed to the rail directly [19], but our sensing setup has the advantage that existing signal cables containing an unused optical fiber can be re-purposed for sensing at no additional cost or installation effort. The measurement stretch is a ballastless track leading to good acoustic coupling between rail and concrete slabs [20]. However, the cable tunnel is not part of this concrete slab structure but lies decoupled from this within soil. There are stretches along the railway where the fiber optic cable is, for example, crossing beneath the tracks or has coils of extra fiber length in certain positions that affect the DAS sensing signal as discussed below. 

We used the commercial Helios DAS system from Fotech Solutions in our experiment. We found a sample rate of 2.5 kHz with pulse widths of 100 ns and a sampling interval of 0.68 m per bin to provide an observable signal. To reduce noise in the data and to make the large datasets of ~10 GB per minute more manageable, we averaged 16 temporal samples for an effective sample time of 6.4 ms or a sampling rate of 156 Hz. After this averaging, typically the signal-to-noise ratio was above 10 over the first 20 km, except for certain faded fiber sections with a lower signal-to-noise ratio. We analyzed only the first 20 km of the 35 km stretch due to the stronger signal levels for shorter distances. However, with different smoothing and thresholding settings, the farther distances can also be analyzed, albeit with increased analysis error.

## 3. Results

### 3.1. Temporal ‘Train-View’ Analysis

**Figure 2:**
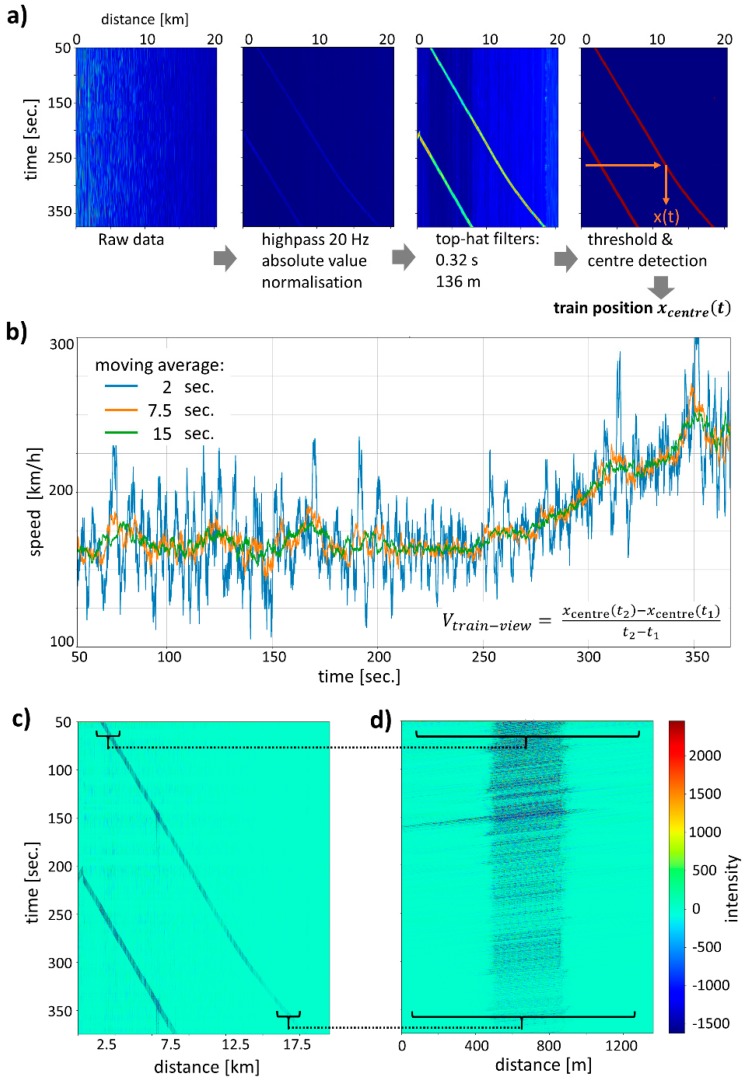
(**a**) The train position *x*_center_*(t)* as a function of time is determined by applying the shown filtering. (**b**) From this position, the train velocity of the ICE 4 train can be calculated at each given moment in time. (**c**) Using the previously determined position of the train, a section of DAS data with the train in the center can be cut and arranged to arrive at the ‘train-view’ representation of the data in (**d**).

A natural way to analyze the time and position-dependent DAS signal *f(x,t)* is to determine the position *x_center_(t)* of the train for each point in time, as shown in Figure 2a. Once the train position *x_center_(t)* is known, the velocity at each moment in time can be calculated as a numerical derivative (Figure 2b). The determination of the train position requires significant data treatment because the raw DAS data is subject to drifts and measurement noise despite the binning of spatial and temporal samples. We have heuristically arrived at the following data processing steps for an optimized determination of the train position. Firstly, we use a 20 Hz high pass filter to remove slower drifts of the data and to normalize the data, so the signals from short and long measurement distances have similar amplitudes. We note that this of course increases the noise floor for measurements at larger distances. Secondly, we take the absolute value and use a top-hat filter with a width of 50 temporal samples (0.32 s in total) to smooth in the temporal direction and a further top-hat filter with a width of 200 bins (136 m) to smooth the spatial direction. In the third step, a threshold of three times the standard deviation of the data was used to create a binary image distinguishing the train and the background. From this band of high signal, the center position *x_center_(t)* of the train was determined using a triangular filter and maximum search. Note that the raw data contain two trains, but the analysis of only one is shown in the following. 

The train velocity calculated from the *x_center_(t)* data is shown in Figure 2b with different temporal averaging applied. The train moves at a constant velocity at first and then accelerates as is already evident from the bent line in the data of Figure 2a. For a 2 s smoothing interval, there is significant noise in the velocity values with a standard deviation of 24 km/h and maximum deviations up to several 100 km/h. Using longer moving average intervals of, e.g., 15 s, the measurement noise is reduced to a standard deviation of 4.5 km/h. Note, that there are several steps in the velocity graph which are caused by the fiber arrangements, such as a fiber crossing beneath the tracks or a coil of extra fiber. All these different fiber geometries result in anomalies in the velocity graph of Figure 2b. For example, a long extra coil of fiber which is passed nearly instantly is registered as a section with unrealistically high velocity. These velocity steps in the signal also cause errors in a moving average and therefore the smoothing of train-view signals in real applications needs to exclude problematic fiber sections. 

Beyond determining the train position and velocity, counting the axles is possible with the DAS data by counting peaks in the signal corresponding to an axle. In our case, due to the spatial resolution limitations in our DAS technique, we performed a measurement where not axles but bogie clusters of the train were counted. While there are individual time samples where the correct number of bogies are visible in the measured intensity along the fiber, in general, the signal is too noisy to determine the correct number from a single sample. Therefore, we use the train position as obtained in the previous step to shift all DAS time samples by *x_center_(t)* to create a ‘train-view’ diagram [6] in which the train and bogie peaks are always localized at the same position (see Figure 2c,d). The train-view diagram corresponds to the DAS signal after transformation into a reference frame moving with the train, and one can directly see the train length of ca. 200 m over time in the diagram. The ‘train-view’ may potentially be useful for detecting train defects via train signals that change over time. After shifting the data, we found that DAS peaks from bogies still do not necessarily lie directly on top of each other. Consequently, we used a second peak finder algorithm looking for peaks in an interval of −10 to 10 m around the center of the train where we expect one bogie cluster to establish the exact position in each time sample. Once these small corrective shifts are applied to the ‘train-view’ image, faint vertical stripes become visible (Figure 2d) corresponding to bogies or bogie clusters. Note that, in principle, the axle/bogie cluster count can be obtained in short time intervals from a train-view representation of the data if only a few samples of sufficient signal quality are averaged. However, lower noise in the data would be desirable and therefore true-phase DAS systems should be used for such an application.

### 3.2. Spatial ‘Rail-View’ Analysis

**Figure 3:**
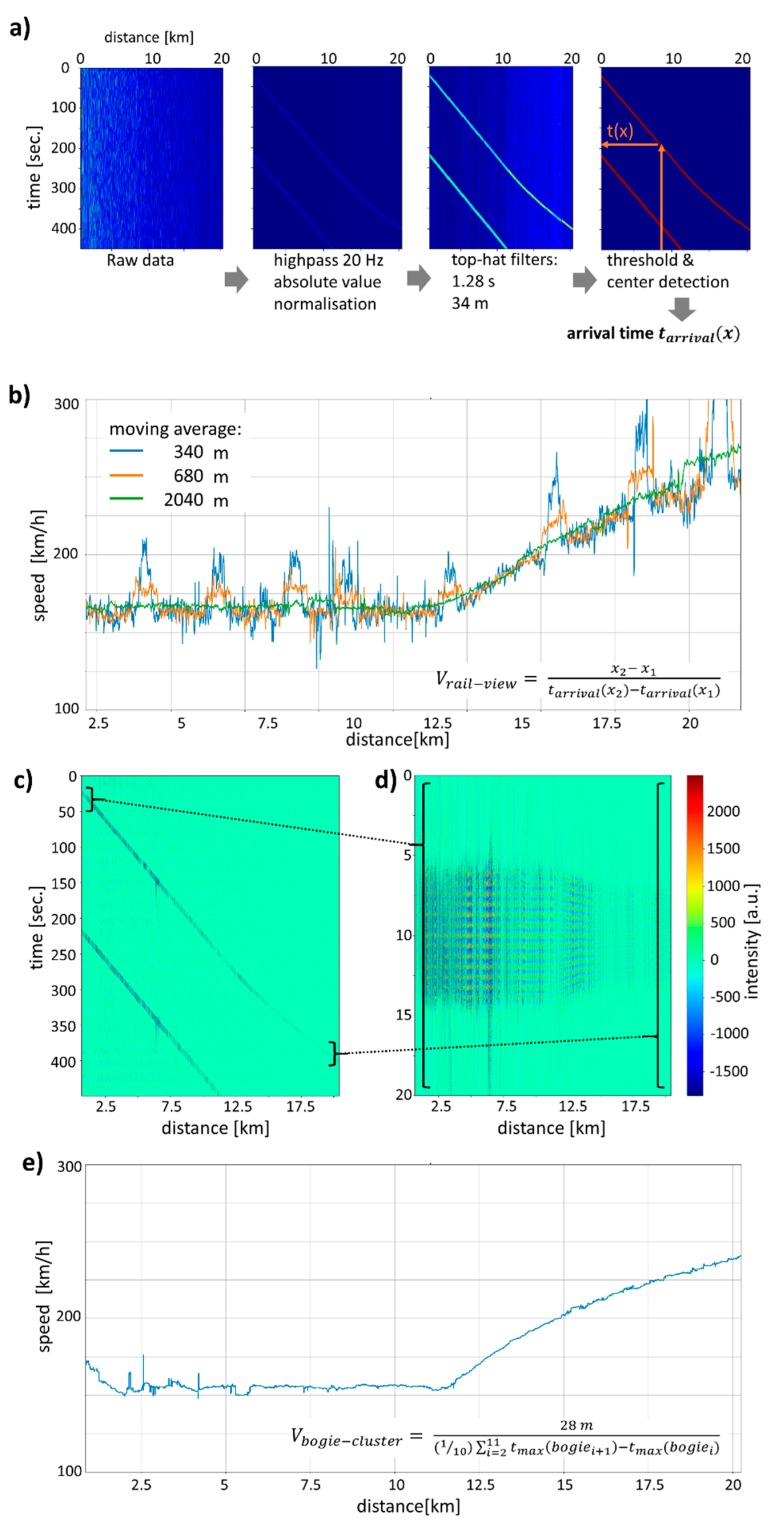
‘Rail-view’ analysis of the DAS data at fixed positions as a function of time. (**a**) Filtering and center detection are similar to Figure 2a, however, the train arrival time *t_arrival_(x)* is determined here. (**b**) From the rail-view data, the train speed is calculated and shown for different averaging lengths. Peaks in the velocity are due to fiber loops. (**c**,**d**) Arranging the 20 Hz filtered data in (**c**) such that the arrival times are aligned results in a ‘rail-view’ plot (**d**). From the distance of the bogie cluster stripes in (**d**), the bogie cluster velocity is determined (**e**).

A second and different possibility to analyze the DAS data is the determination of the arrival time *t_arrival_(x)* of the train at a given fiber/rail position x. This is different from the above analysis of *x_center_(t)* because it avoids the problem of fading, that is the low signal strength for certain positions in x along the fiber. This randomness of the DAS signal from different positions is modulating the train signal and makes finding the train center as performed above difficult. However, the random fading signal in our measurement signal at a given position is varying smoothly during the passage time of the train so that no significant fluctuation obscures the train signal. Consequently, determining the time *t_arrival_(x)* when the train center passes a certain position *x* is making use of a cleaner signal. Obviously, one needs to wait until the whole train has passed a certain position and therefore the algorithm can only give updates after the time it takes the train to pass a certain position. As a consequence, there is a trade-off between updates after very short intervals possible with the train-view analysis above and the slower ‘rail-view’ analysis with better signal-to-noise ratio as shown in Figure 3.

The data treatment is similar to the one from Figure 2, which is a 20 Hz high pass filter, top-hat filters in the temporal and spatial direction followed by thresholding for selecting the train. Finally, the arrival time *t_arrival_(x)* of the train at each position is calculated using center detection via a triangular filter and finding its maximum. Again, the train velocity can be calculated from the time and position pairs, and the result is shown in Figure 3b for different spatial averaging. For a moving average of 341 m and approximately 5 km distance from the DAS instrument, the standard deviation of the velocity values is 4.8 km/h when the train is moving at 160 km/h. This standard deviation does not include sections with up to 70 km/h deviations, which are due to extra fiber length next to the track. As discussed above, the coils found in regular intervals next to the track result in false velocity estimates. The noise and therefore velocity uncertainty increase with distance from the measurement unit, but also with train speed toward the right in our example. For an estimate of the velocity-dependent standard deviation of train velocities, different trains moving constantly at 160 km/h and 250 km/h (not shown) were analyzed in the same fiber interval and the standard deviations were 4.8 and 7.2 km/h, respectively.

Similar to the ‘train-view’ image above, we can construct a ‘rail-view’ image by shifting the train signal in time such that the arrival time of the train center is displayed at a fixed time point (see Figure 3d). The points *t_arrival_(x)* are not precise enough to resolve a stripe pattern of the bogie cluster passing by within the train signal. Therefore, we again used peak detection within an interval corresponding to the passage time of one car and perform a fine shift in the temporal direction to align the train arrival times. The result of this procedure is shown in Figure 3d; clearly, red and blue horizontal stripes can be seen. Each of the red stripes corresponds to a maximum intensity due to a bogie/bogie cluster of the train. The 13 bogie clusters of the ICE 4 train can be discerned in most sections of the fiber apart from a few sections affected by fading or low signal due to bad acoustic coupling. In contrast to the train-view, which always displays the complete length of the train, the time interval of the rail-view representation depends on the velocity of the train and the stripe pattern is wider for the lower velocity on the left and narrower for higher velocities where the passage time is reduced. In conclusion, the rail-view of Figure 3d is more adequate for counting bogie clusters than the train-view graph of Figure 2d because the bogie signals can be aligned more precisely, are more prominently visible, and therefore can be averaged to obtain reliable counts of bogie clusters. From the rail-view signal in Figure 3d, the number of bogie clusters can be counted as 13.0 ± 0.4 for an averaging interval of 1.36 km, which is roughly every one to two km the train integrity can be established by the Fotech DAS system.

### 3.3. Artificial Neural Network (ANN) Analysis

**Figure 4:**
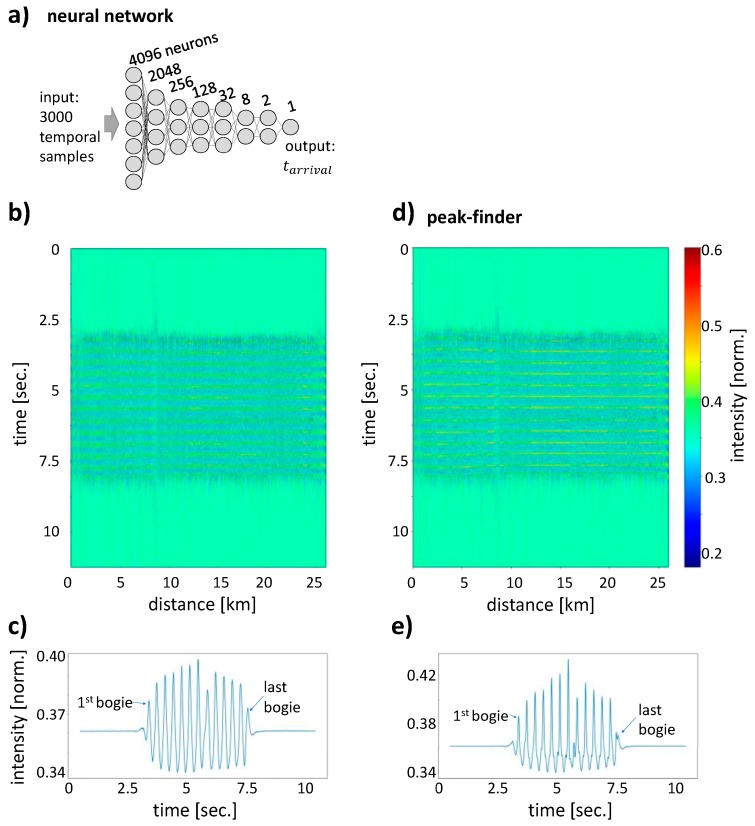
(**a**) Artificial neural networks (ANN) can successfully predict the precise arrival times of a bogie cluster so that a well aligned rail-view graph (**b**) can be generated. (**c**) Taking the spatial average of the rail-view graph, all bogie clusters can clearly be resolved as 13 peaks. A peak finder algorithm performs similarly to the ANN in aligning the bins for a rail-view graph (**d**) and again 13 peaks can be found as well but the peak height is more uneven (**e**).

We have tested a range of algorithms to align the DAS signals and ANN have been found to work reliably and quickly on the large datasets. Both the ANN and the peak finder have been applied not to the complete dataset but to the data after the above rough train localization procedure. The additional alignment after a more precise determination of the time *t_arrival_(x)* makes the stripe pattern due to bogies visible in a train-view graph. The ANN consisted of an input layer where the signal of 3000 temporal samples containing the train signal at a fixed spatial position *x* is handed to the dense network. This is followed by seven hidden layers of 4096, 2048, 256, 128, 32, 8, and 2 neurons with relu activation functions apart from the last layer with linear activation functions. The single output variable corresponds to *t_arrival_(x)*, which is the time shift necessary to align the bogies at precisely the same time (see Figure 4a). The overall network architecture is not critical and a different number of neurons or shallower networks trained and performed similarly. The ANN was presented with DAS data normalized at every position as training data. To get a larger training dataset, we also created synthetic DAS data where we computationally shifted the train signal in time to get more examples at different known temporal shifts. Using this training dataset of in total 96,000 examples, we performed an optimization of the weights connecting neurons in the Keras and TensorFlow framework for 20 iterations (epochs).

The results of the position determination and alignment with the ANN model in Figure 4a are shown in Figure 4b,c and compared with the peak finder algorithm in (Figure 4d,e). The data shown is not the ICE 4 train signal used for training but data from a similar ICE 4 train passing two hours later. The rail-view stripe pattern in the DAS data is clearly visible for both algorithms. Figure 4c,e show line graphs that result from spatially averaging the entire rail-view plots. The first and last bogie are more clearly visible in the ANN data (Figure 4c), while the peaks are more pronounced in the data output of the peak finder Figure 4e. Note that the high middle peak followed by a lower peak to the right is an artifact of the peak finder algorithm. These higher and lower peaks are visible also in the ANN analysis, because data aligned with the peak finder has been used as training data. Despite this use of the peak finder algorithm to train the ANN, the ANN generalizes the training data so that their results are non-identical and the ANN output is more suitable for counting all bogie clusters. The peak finder results in narrower peaks where even some sub-structure, potentially due to individual axes, is visible. 

The use of the ANN offers advantages in processing speed as well as flexibility to process different datasets. The filtering and localization of the train DAS data with the subsequent alignment of bogie clusters by the peak finder algorithms in the dataset take 300 s. In contrast, coarser DAS data filtering and train localization together with bogie cluster alignment by an ANN takes only 22 s, which is the filtering and ANN processing is more than ten times faster. The speed gain results from the fact that an ANN can align the bogie clusters even if the localization of the train center has deviations of up to ±1 train car, while the peak finder needs the correct, center bogie cluster within its search range of ±0.5 a train car. As a result, the coarser filtering algorithm for the ANN can work on fewer samples and is significantly faster than the filtering for the peak finder algorithm. The peak finder was also optimized to the given train velocity and more complicated filter banks are necessary for processing data from trains at different speeds. Again, the ANN has advantages as it is more flexible when trained with data of trains with changing speed. Further improvements to the ANN model would be possible through training the network with ground truth data about the actual train position for example via global navigation satellite systems instead of data from the peak finding algorithm. 

## 4. Discussion

In the above examples, we have demonstrated how a commercial DAS instrument can be used to detect the positions of the whole train as well as bogie clusters. From these results, the train velocity can be determined in three distinct ways using train-view, rail-view, and bogie cluster data analysis (Figure 2b and Figure 3b,e). In Table 1, we summarize the standard deviation in the velocity determination for our case study. The results show that the bogie cluster velocity has the lowest standard deviation followed by rail-view and lastly train-view velocities. The slight improvement for rail-view in comparison with train-view can be explained by the position-dependent fading, which introduces fluctuations in signal strength and affects the train localization in the spatial direction. For the rail-view analysis at a fixed position of the fiber, fading effects are mostly constant and do not affect the train localization in the time direction. The bogie cluster velocity, which uses the sub-structure in the train noises, improves on the train-view or rail-view velocity precision by more than a factor of four and, in contrast to the other velocities, is not affected by spurious jumps in velocity due to extra fiber length or details of the fiber-track distance and geometry. Note that the erroneous velocity jumps have been removed for the calculation of the standard deviation from train- and rail-view. But, even with these corrections to train-view and rail-view, the bogie cluster velocity is more precise. In the future, also combinations of the three analysis methods are possible for more reliable determination of the velocity. 

The standard deviations given in Table 1 have been determined for a train moving at 160 km/h and depend on the train velocity. According to error propagation, the standard deviation of the velocity is $\delta v=v\cdot \sqrt{\delta t/t^{2}+\delta x/x^{2}}$ and therefore increases linearly with velocity if the position or time errors remain constant. This naïve linear relation will not be strictly fulfilled as the errors in the DAS position/time determination will increase at low velocities where the train generates lower noises and therefore lower DAS signal. At elevated train speeds, we find an increase in the velocity error that is roughly proportional to the velocity. For example, we observe for rail-view velocities a standard deviation of ±4.8 km/h at 160 km/h and 7.2 km/h at 250 km/h (data not shown, both averaged for 340 m). 

It is important to note that all the standard deviations given have been averaged either in time or in space. In time, 7.5 s corresponds to the time it takes a train to pass a certain position at 160 km/h. In space, the train length over all the bogies is 340 m so that 7.5 s or 340 m averaging is comparable. While all velocity measurements can be updated in 6.8 ms intervals, this velocity update does not correspond to the real-time velocity but a previous train velocity, and different analysis and averaging schemes have different delays. In train-view, the velocity calculated for example with 15 s moving average corresponds to the train velocity 15 s prior in the center of the averaging window. In rail-view or bogie cluster velocities, the spatial averaging similarly introduces a delay in the velocity determination. For example, 680 m averaging introduces a 15 s delay at 160 km/h. Importantly, another 7.5 s must be added to this value because, at each position, one must wait for the train to pass for a total delay of 22.5 s. Therefore, both rail-view and bogie cluster velocity have larger delays. Their minimum delay is limited by the train passage time and depends on the train speed. In conclusion, train-view has an advantage for fast real-time monitoring down to a theoretical limit of 6.4 ms but accepting some delay in rail-view and bogie cluster velocities are useful and more precise. 

The data analysis, filtering, and neural network techniques are a field of active development with a range of algorithms such as edge detection by sliding variance, principal component analysis of the train frequency spectrum, or wavelet transformation and are discussed in the literature [11,14]. In real-time monitoring, the computer processing time of the big datasets is a concern so that time-optimized algorithms have been presented, e.g., in reference [15], where wavelet analysis has been replaced by faster smoothing and Canny edge detection filters. ANN processing time is promising in this regard. The ANN is currently only used to locate the train in a short 12 s time interval pre-determined by using a conventional filter algorithm, but the slow pre-processing can potentially be integrated in a much faster ANN data analysis. In conclusion, further optimization of the speed and quality of the raw data processing remains an important task, both for DAS and true-phase DAS systems.

Newer generations of DAS systems, in particular true-phase coherent optical time-domain reflectivity (COTDR) systems, as well as true-phase and low drift wavelength scanning COTDR systems, yield better data quality compared to the one used in this study. Therefore, we expect that the precision demonstrated above can be achieved with less averaging and therefore shorter update intervals and higher spatial resolution even at the meter level for axle counting. A higher signal-to-noise ratio of the data will make it possible to measure at larger distances from the interrogator unit. The bogie cluster velocity will profit from the higher data quality that enables one to resolve each axle and not just bogie clusters as shown here. However, the above observations of analysis strategies using train-view and rail-view representations as well as the possibilities of ANNs for axle/bogie cluster detection with the associated velocity determination remain valid and important also for these better data qualities.

## 5. Conclusions

We have demonstrated that distributed fiber optic sensing with standard telecom fibers can determine current position, velocity, and bogie cluster count during the movement of ICE 4 trains of DB AG. We have shown that a first train-view analysis method is suited for the determination of train position and velocity. A second, slightly slower rail-view analysis is less susceptible to fluctuations of the fiber scattering and results in lower velocity uncertainty. Importantly, this rail-view analysis together with peak finder or artificial neural network algorithms makes it possible to resolve individual bogies or bogie clusters in the signal so that the train cars can be counted and train integrity can be monitored. From the bogie signal and the time between the bogie passage, a velocity can be calculated with an uncertainty of down to ±0.8 km/h depending on averaging length and time. Our work further demonstrates that training artificial neural networks with past train data can be used to analyze future train movements and is more than ten times faster and can handle more varied input better than our conventional algorithm. In the future, work is needed to investigate the velocity uncertainties of slower-moving trains on older rail infrastructure. While initial tests validate the analysis approach, the data quality is significantly lower and true-phase DAS systems may be required to achieve higher signal-to-noise ratios in the data. In conclusion, this quantitative study opens ways for train monitoring as well as more intricate analysis for example of train or rail defects via DAS. 

## Figures and Tables

**Table 1:** Standard deviation of the velocity for train tracking of an ICE 4 train moving at 160 km/h using different signal processing and averaging intervals.

$\delta v_{train-view}$	$\delta v_{rail-view}$	$\delta v_{bogie-cluster}$
±24 km/h (avg. 2 s)	-	-
±5.1 km/h (avg. 7.5 s)	±4.8 km/h (avg. 341 m)	±1.2 km/h (avg. 341 m)
±4.5 km/h (avg. 15 s)	±3.5 km/h (avg. 681 m)	±0.8 km/h (avg. 681 m)
